# Revision of the paraphyletic genus *Koerneria* Meyl, 1960 and resurrection of two other genera of Diplogastridae (Nematoda)

**DOI:** 10.3897/zookeys.442.7459

**Published:** 2014-09-23

**Authors:** Natsumi Kanzaki, Erik J. Ragsdale, Robin M. Giblin-Davis

**Affiliations:** 1 Forest Pathology Laboratory, Forestry and Forest Products Research Institute, 1 Matsunosato, Tsukuba, Ibaraki 305-8687, Japan; 2 Fort Lauderdale Research and Education Center, University of Florida/IFAS, 3205 College Avenue, Davie, FL 33314-7719, USA; 3 Department of Biology, Indiana University, 1001 E. 3rd Street, Bloomington, IN 47405, USA

**Keywords:** *Allodiplogaster*, *Anchidiplogaster*, *Koerneria*, *Pristionchus*, revision, phylogeny, taxonomy

## Abstract

Recent inferences of phylogeny from molecular characters, as well as a reexamination of morphological and biological characters, reject the monophyly of the nematode genus *Koerneria* Meyl, 1960 (Diplogastridae). Here, *Koerneria* sensu lato is revised. The genus, which previously consisted of 40 species, is separated into three genera. Almost all of the transferred species are moved to the resurrected genus *Allodiplogaster* Paramonov & Sobolev in Skrjabin et al. (1954). *Koerneria* and *Allodiplogaster* are distinguished from each other by a weakly vs. clearly striated body surface, an undivided vs. divided stomatal cheilostom, and arrangement of the terminal ventral triplet of male genital papillae, namely in that v5 and v6 are paired and separated from v7 vs. v5–v7 being close to each other. *Allodiplogaster* is further divided into two groups of species, herein called the *henrichae* and *striata* groups, based on both morphological and life-history traits. The *henrichae* group is characterized by papilliform labial sensilla and male genital papillae, a conical tail in both males and females, and an association with terrestrial habitats and insects, whereas the *striata* group is characterized by setiform labial sensilla and male genital papillae, an elongated conical tail in both sexes, and an association with aquatic habitats. A second genus, *Anchidiplogaster* Paramonov, 1952, is resurrected to include a single species that is characterized by its miniscule stoma and teeth, unreflexed testis, and a distinct lack of male genital papillae or stomatal apodemes. Lastly, one further species that was previously included in *Koerneria* sensu lato is transferred to the genus *Pristionchus* Kreis, 1932. The revision of *Koerneria* sensu lato is necessitated by the great variability in its subordinate taxa, which occupy a variety of habitats, in addition to the increased attention to Diplogastridae as a model system for comparative mechanistic biology.

## Introduction

*Koerneria* Meyl, 1960 heretofore consisted of 40 nominal species, following the revision by Sudhaus and Fürst von Lieven (2003) and including species described since then (Suppl. material 1). Several unidentified or undescribed species with molecular vouchers have also been reported (Mayer et al. 2007). Biological characters are variable for the genus, which contains terrestrial species isolated from rich soil environments, associates of several different groups of insects, and aquatic species. Such a diversity of ecologies raises the question of whether distinct subgroups of the genus can be identified and corroborated by independent characters.

**Figure 1.**
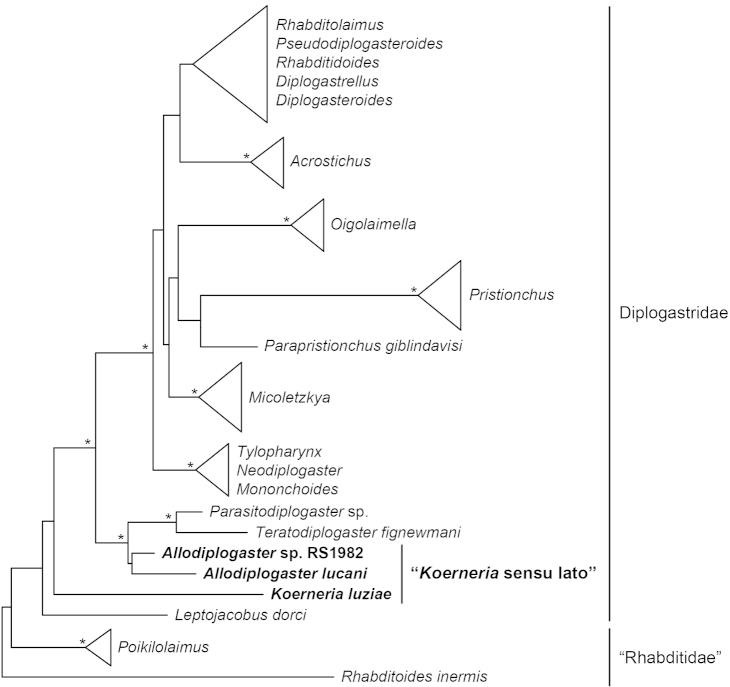
Paraphyly of *Koerneria* sensu lato. Tree is simplified from Kanzaki et al. (2014b), which was inferred from nearly full-length small subunit ribosomal DNA sequences. A subsequent study that included several more species of *Koerneria* sensu lato likewise showed the well-supported exclusion (99% bootstrap support in likelihood analysis, 100% posterior probability in Bayesian analysis) of a clade of *Koerneria luziae* + *Koerneria ruehmi* from all other Diplogastridae, including a monophyletic clade of what is designated herein as *Allodiplogaster* (Atighi et al. 2013). Asterisks indicate nodes with very strong support as inferred in the former study (>95% bootstrap support, 100% posterior probability).

In their revision of Diplogastridae, Sudhaus and Fürst von Lieven (2003) circumscribed *Koerneria* by the following characters: (1) presence of stomatal dimorphism, (2) cheilostom often separated into six per- and interradial figs or small stick-like figs (= rugae), (3) vertical striation, (4) stegostom with a dorsal claw-like tooth, a right subventral tooth, and left subventral serrated figs, (5) postdental region of stegostom with two subventrad directed apodemes, (6) female gonad amphidelphic or seldom prodelphic, and (7) no bursa. Later, Fürst von Lieven (2008) transferred *Koerneria colobocerca* (Andrássy, 1964) Fürst von Lieven, 2008 from *Mononchoides* Rahm, 1928 and revised the generic definition by adding two characters, (8) intestine sometimes with a prerectum, and (9) tail filiform or conical. Given the breadth of the most recent morphological definition of the genus, the only absolute generic character is the presence of articulated apodemes in the stoma. However, phylogenetic studies of molecular characters strongly indicate that the genus, as currently interpreted, is paraphyletic and thus separable into two or more clades (Fig. 1). Therefore, a taxonomic reorganization of *Koerneria* sensu lato (= *Koerneria* sensu Fürst von Lieven 2008) is needed. Such a revision is of particular importance for ongoing studies of the natural history of the genus and its relatives (e.g., Giblin-Davis et al. 2006, Kanzaki et al. 2009) as well as comparative research more generally in Diplogastridae (e.g., Mayer et al. 2009, Ragsdale et al. 2013), as *Koerneria* sensu lato includes taxa that are sister groups to most other known species in the family.

In this article, we revise the genus *Koerneria* by examining the original and subsequent descriptions of its nominal species. Based on morphological, biological, and molecular evidence, we separate *Koerneria* sensu lato into three genera. All renamed groups are hypothesized to be monophyletic and follow the precedent of previous classification systems (Andrássy 1984). Besides limiting the scope of *Koerneria*, we resurrect the genera *Anchidiplogaster* Paramonov, 1952 and *Allodiplogaster* Paramonov & Sobolev in Skrjabin et al. (1954). Furthermore, we distinguish two putative clades of *Allodiplogaster* species, each of which we hypothesize to be monophyletic on available information, although we leave formal revision of this genus to follow molecular studies of unsampled aquatic taxa.

### Materials and methods

Species of *Koerneria* sensu lato are classified herein into four typological groups (three genera, with one genus further separated into two morphological and ecological groups) based on the following characters or traits, which were selected due to their high availability and reliability, being relatively accurate even in old descriptions:

(1) Stomatal morphology, specifically the separation of cheilostom and presence of apodemes

(2) Male tail morphology, including shape and arrangement of genital papillae

(3) Female tail morphology

(4) Life-history characters, particularly habitat preferences

In addition to published literature, several species available in culture were examined for typological characters: *Koerneria luziae*, isolated from stag beetles from Japan (Kanzaki et al. 2011); *Koerneria* sp., isolated from *Dorcus rectus* (Coleoptera: Lucanidae) from Japan; *Allodiplogaster* spp. RGD227 and RGD228, both isolated from soil-dwelling bees in the United States. Stomatal morphology and tail characters were examined by differential interference contrast (DIC) microscopy according to methods described by Kanzaki (2013). Several schematic illustrations were prepared based on original observations as well as published data. Following the reexamination of informative characters, the original descriptions and revisions were reviewed to determine the generic status of species following the International Code of Zoological Nomenclature (ICZN).

## Systematics

Previous inferences of the phylogeny of *Koerneria* spp. indicate that two species, *Koerneria luziae* (Körner, 1954) Meyl, 1960 and *Koerneria ruehmi* Atighi, Pourjam, Kanzaki, Giblin-Davis, De Ley, Mundo-Ocampo & Pedram, 2013, form a well-supported clade that is the sister group to most or all other sequenced Diplogastridae (Atighi et al. 2013; Kanzaki et al. 2014b; Fig. 1). Separate from this group is a well-supported clade consisting of *Koerneria lucani* (Körner, 1954) Meyl, 1960 and some unidentified or undescribed species (Fig. 1). The former two species share two features that clearly distinguish them from other species of *Koerneria* sensu lato, namely (1) a tube- or ring-like (undivided) cheilostom and (2) male genital papillae arranged such that v5 and v6 (papillae nomenclature follows Sudhaus and Fürst von Lieven 2003) are close to each other and v7 is clearly apart from v6. These two characters diagnose six nominal species of *Koerneria* sensu lato, including the above two as well as the type species of the genus. We therefore revise the genus as follows:

### *Koerneria* Meyl, 1960

Fig. 2

**Figure 2.**
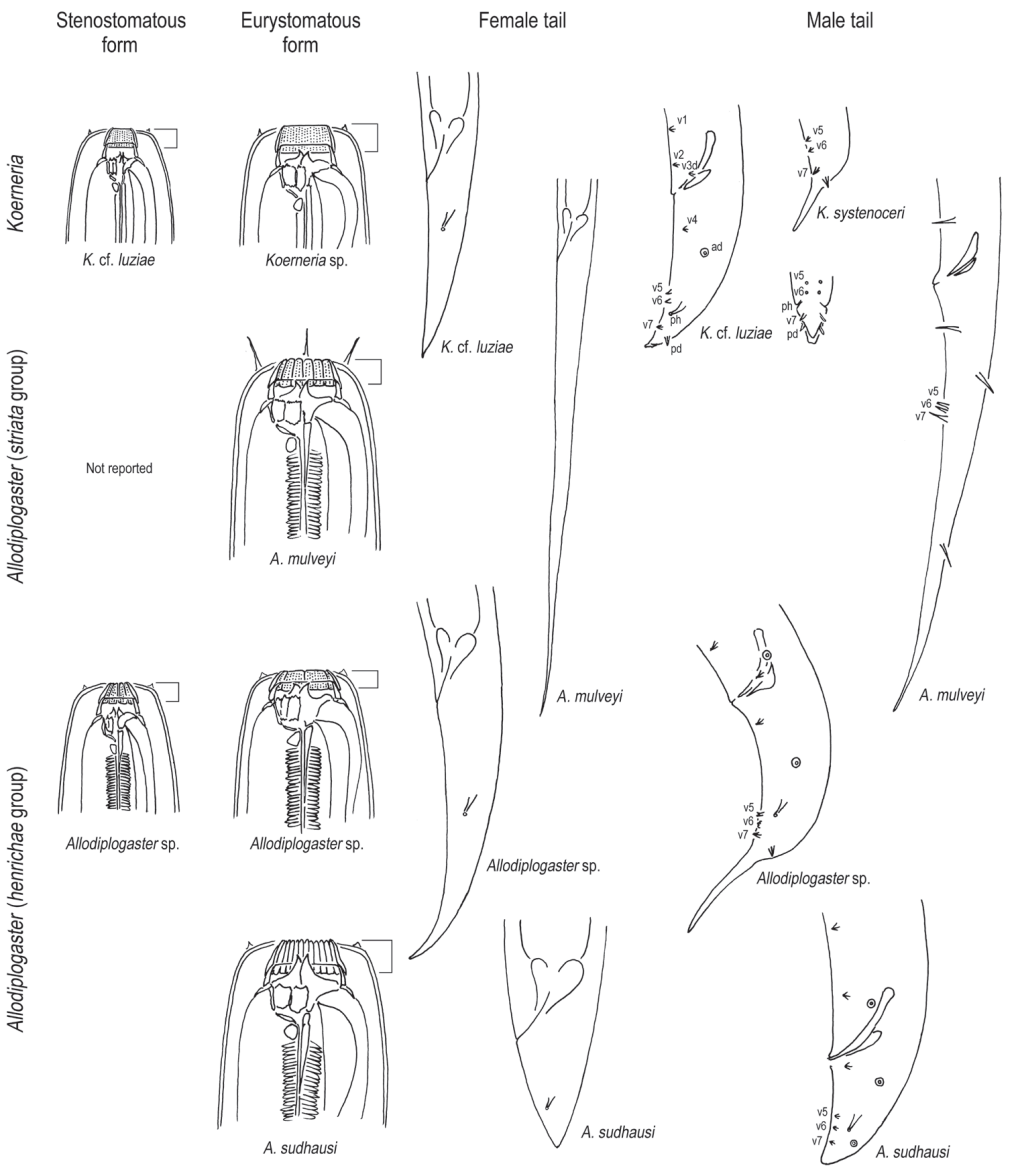
Schematic drawings of the generic characters of *Koerneria* and *Allodiplogaster*. From left to right: stenostomatous form, eurystomatous form, female tail, and male tail characters. From top to bottom: *Koerneria*, aquatic *Allodiplogaster* (“*striata*” species group) and two types of terrestrial *Allodiplogaster* (“*henrichae*” species group). For the stenostomatous form of *Allodiplogaster sudhausi* (not shown), see Fürst von Lieven (2008). Squared bracket indicates cheilostom, which as undivided separates *Koerneria* from *Allodiplogaster*. Further diagnosing *Koerneria* is the arrangement of male genital papillae v5-v7. Unique to the *striata* group of *Allodiplogaster* relative to the *henrichae* group and to *Koerneria* are a long tail in both sexes, setiform genital papillae, and in many cases setiform labial papillae. Genital papillae and phasmids are labeled following the terminology in Sudhaus and Fürst von Lieven (2003). The phasmids are not clearly described in species of the *striata* group.

**Generic diagnosis**

1) Stomatal dimorphism occasionally present^1^

2) Body-wall cuticle with weak vertical striations

3) Cheilostom usually forming short, undivided tube; rugae absent

4) Stegostom with dorsal claw-like tooth, right subventral tooth, and left subventral serrated figs or ridges

5) Postdental region of stegostom with left and right apodemes directed subventrad

6) Female gonad amphidelphic

7) Anterior two ventral and distal pairs of genital papillae (v5 and v6) close to each other, the posterior pair (v7) being clearly apart from v6

8) Short and conical male tail (c’ is usually ≤ 3) usually with short spike or with small, bursa-like or membranous appendage at tail tip

9) Known from terrestrial habitats, often in association with insects

**Type species**

*Diplogaster goffarti* Körner, 1954

comb. *Koerneria goffarti* Meyl, 1960

**Other species**

*Koerneria erlangensis* (Sachs, 1950) Sudhaus & Fürst von Lieven, 2003

*Koerneria luziae* (Körner, 1954) Meyl, 1960

*Koerneria ruehmi* Atighi, Pourjam, Kanzaki, Giblin-Davis, De Ley, Mundo-Ocampo & Pedram, 2013

*Koerneria sinodendroni* (Körner, 1954) Meyl, 1960

*Koerneria systenoceri* (Körner, 1954) Meyl, 1960

Following this restricted definition of *Koerneria*, most of the remaining species of *Koerneria* sensu lato are transferred to the resurrected genera *Anchidiplogaster* and *Allodiplogaster*. One species, which was previously combined as *Koerneria dubia* (Hnatewytsch, 1929) Sudhaus & Fürst von Lieven, 2003 is returned to *Anchidiplogaster* based on a suite of characters unique to this taxon as well as by the lack of stomatal apodemes, an absolute character of *Koerneria* as defined both previously and herein:

### *Anchidiplogaster* Paramonov, 1952

**Generic diagnosis**

1) Miniscule, undivided stoma with two small, similarly sized pyramidal teeth (one dorsal and one right subventral)

2) Stomatal apodemes absent

3) Male genital papillae absent

4) Testis without flexure

**Type and only species**

=*Diplogaster dubia* Hnatewytsch, 1929

comb. *Anchidiplogaster* Paramonov, 1952

All but one of the remaining species of *Koerneria* sensu lato are transferred to the other resurrected genus, *Allodiplogaster*. This name has priority (ICZN 23.1) over other names that are available for this taxonomic grouping, which consist of *Diplenteron* Andrássy, 1964, *Glauxinemella* Gagarin, 1998, and *Gobindonema* Khera, 1970. Furthermore, *Allodiplogaster* is separated into two putatively monophyletic groups of species, which we designate as the “*henrichae* group” and “*striata* group” based on morphological and biological evidence.

### *Allodiplogaster* Paramonov & Sobolev in Skrjabin, Shikobalova, Sobolev, Paramonov & Sudarikov, 1954

Fig. 2

=*Diplenteron* Andrássy, 1964: *Diplenteron colobocercus* Andrássy, 1964

=*Gobindonema* Khera, 1970: *Gobindonema filicaudata* Khera, 1970

*nec Gobindonema* Sood & Prashad, 1974 (Trichostrongylidae)

=*Glauxinemella* Gagarin, 1998: *Glauxinemella striata* Gagarin, 1998

**Generic diagnosis**

1) Stomatal dimorphism occasionally present

2) Body-wall cuticle with clear vertical striations

3) Cheilostom separated into six per- and interradial figs or rugae

4) Stegostom with dorsal claw-like tooth, right subventral tooth, and left subventral serrated figs or ridges

5) Postdental region of stegostom with left and right apodemes directed subventrad

6) Female gonad amphidelphic; rarely prodelphic

7) Distal triplet papillae of males (v5-7) close to each other

8) Tail of male and females highly variable in shape

9) Known from variable habitats including terrestrial insect associates and aquatic species

**Type species**

*Diplogaster henrichae* Sachs, 1950

comb. *Allodiplogaster henrichae* Paramonov & Sobolev in Skrjabin, Shikobalova, Sobolev, Paramonov & Sudarikov, 1954

**Other species**

***henrichae* group of *Allodiplogaster***

1) Labial sensilla usually papilliform

2) Female tail usually conical with or without filiform tip

3) Male tail usually conical with short spike

4) Male genital papillae short and papilliform

5) Known from terrestrial habitats, often in association with insects

*Allodiplogaster colobocerca* (Andrássy, 1964), comb. n.

=*Mononchoides potohikus* Yeates, 1969

*Allodiplogaster hirschmannae* (Sachs, 1950), comb. n.

*Allodiplogaster histophora* (Weingärtner, 1955), comb. n.

*Allodiplogaster hylobii* (Fuchs, 1915), comb. n.

*Allodiplogaster incurva* (Körner, 1954), comb. n.

*Allodiplogaster labiomorpha* (Kühne, 1995), comb. n.

*Allodiplogaster lepida* (Andrássy, 1958), comb. n.

*Allodiplogaster lucani* (Körner, 1954), comb. n.

*Allodiplogaster pierci* (Massey, 1967), comb. n.

*Allodiplogaster pini* (Fuchs, 1931), comb. n.

*Allodiplogaster robinicola* (Rühm, 1956), comb. n.

*Allodiplogaster sudhausi* (Fürst von Lieven, 2008), comb. n.

***striata* group of *Allodiplogaster***

1) Labial sensilla setiform

2) Male and female tail usually elongate-conical, with or without filiform tip

3) Male genital papillae setiform

4) Known from aquatic habitats

*Allodiplogaster angarensis* (Gagarin, 1983), comb. n.

*Allodiplogaster aquatica* (Dassonville & Heyns, 1984), comb. n.

*Allodiplogaster baicalensis* (Tsalolichin, 1972), comb. n.

*Allodiplogaster carinata* (Zullini, 1981), comb. n.

*Allodiplogaster didentata* (Hnatewytsch, 1929), comb. n.

=*Diplogaster curvidentatus* Altherr, 1938

=*Diplogaster obscuricola* Altherr, 1938

=*Diplogaster quadridentatus* Altherr, 1938

*Allodiplogaster filicaudata* (Khera, 1970), comb. n.

*Allodiplogaster ivanegae* (Gagarin, 1983), comb. n.

*Allodiplogaster lupata* (Shoshin, 1989), comb. n.

*Allodiplogaster mordax* (Shoshin, 1989), comb. n.

*Allodiplogaster mulveyi* (Ebsary, 1986), comb. n.

*Allodiplogaster pantolaba* (Shoshin, 1989), comb. n.

*Allodiplogaster pararmata* (Schneider, 1938), comb. n.

=*Diplogaster armatus apud* Filipjev, 1930, *nec* Hofmänner, 1913

*Allodiplogaster regia* (Shoshin, 1989), comb. n.

*Allodiplogaster ruricula* (Gagarin, 1983), comb. n.

*Allodiplogaster sphagni* (Soós, 1938), comb. n.

*Allodiplogaster strenua* (Gagarin, 1983), comb. n.

*Allodiplogaster striata* (Gagarin, 1998), comb. n.

*Allodiplogaster tenuipunctata* (Altherr, 1938), comb. n.

*Allodiplogaster terranova* (Ebsary, 1986), comb. n.

The single remaining species previously included in *Koerneria* sensu lato is transferred to *Pristionchus* Kreis, 1932. This transfer is supported by the absence of subventral apodemes, the key character diagnosing *Koerneria* sensu lato:

*Pristionchus macrospiculum* (Altherr, 1938), comb. n.

## Discussion

The separation of *Koerneria* as circumscribed herein from *Allodiplogaster*, *Anchidiplogaster*, and *Pristionchus* is strongly supported by the structure of the cheilostom, arrangement of male genital papillae, and phylogeny as inferred independently from molecular sequence characters (Atighi et al. 2013; Kanzaki et al. 2011, 2013, 2014b). Although for the previous, wider definition of *Koerneria* the name *Koerneria* was itself a junior synonym of *Allodiplogaster*, the name *Koerneria* is retained for a group of six described species, all of which are unambiguously unified by morphological characters and which are represented by a clade not nested within any other valid genus. Furthermore, species of *Koerneria* in the revised sense are apparently the sister group to most or all species of Diplogastridae, with the possible one exception of *Leptojacobus dorci* (see Kanzaki et al. 2014b), and therefore the revision of this genus will be useful for ongoing research on the family as a comparative model system.

The distinct morphology of *Anchidiplogaster dubia*, especially its lack of genital papillae in several observed male specimens separates this species from all other Diplogastridae and supports its reestablishment in that genus. Further indicating the distinctness of this species from most other known Diplogastridae is its unreflexed testis flexure, as a flexure was previously considered a plesiomorphic character of the entire family (Sudhaus and Fürst von Lieven 2003, Andrássy 2005), although one other diplogastrid genus, *Leptojacobus* Kanzaki, Ragsdale, Susoy & Sommer, 2014, has since been described to have no flexure. In the original description of *Anchidiplogaster dubia*, the above missing features were explicitly given as diagnostic characters (Hnatewytsch 1929) and thus were unlikely to be simply missed in all specimens examined. An undivided stoma is also unusual among most diplogastrids with subventral teeth, although this type of stoma is present in *Koerneria* in the revised sense and is thus insufficient in itself to diagnose *Anchidiplogaster*. The presence of asymmetrical teeth and a glandular postcorpus clearly support its identity as a diplogastrid, the otherwise divergent stomatal morphology in this species, namely its diminutive, undivided stomatal cavity armed with triangular teeth, obscure its relationships to other taxa within the family. Because molecular characters are not available for this species, its position in Diplogastridae cannot be predicted with any certainty.

The split of *Allodiplogaster* into the *henrichae* and *striata* groups is also supported by morphology, principally by the tail of both sexes, which is usually much longer in *striata* group than in *henrichae* group, and by the male genital papillae and labial sensilla, which are distinctly setiform in *striata* group. The separation of the two proposed groups of *Allodiplogaster* is only confounded by overlapping morphological characters, namely the fish-bone-like swellings along the pharyngeal lumen that are present in *henrichae* group species, *Allodiplogaster* spp. RGD227 and RGD228, as well as in one species of the *striata* group, *Allodiplogaster carinata*, although not in another *striata* group species such as *Allodiplogaster pararmata* (Fürst von Lieven 2001, Fürst von Lieven and Sudhaus 2000, Kanzaki et al. unpubl. obs.). However, no species of the *striata* group have been molecularly characterized thus far, so the phylogenetic position of that group has yet to be tested by gene sequence data. Reisolation of species, particularly of the *striata* group, will be necessary for fully testing the system of intrageneric grouping presented here. Complete taxonomic revision of *Allodiplogaster* is therefore not performed here due to a lack of molecular evidence, although further studies may confirm the separation of the two species groups designated here into different genera.

Other morphological characters may further support to separate *henrichae* group from as a monophyletic clade distinct from the *striata* group and from *Koerneria*. Kühne (1995) examined three *henrichae* group species by scanning electron microscopy and reported important morphological features of the male tail: the modification in v5 and v6 subventral distal papillae, whereby v5 is rooted in a socket-like base and has split tip, and v6 having two anterior and posterior appendages and being likewise rooted in a socket-like base (Fig. 2). This morphology, which is similar to the trifurcate genital papillae of *Diplogasteroides* spp. (Kiontke et al. 2001, Kanzaki et al. 2013), is otherwise unique among Diplogastridae and Rhabditidae thus far characterized (e.g., Kiontke and Sudhaus 2000, Kanzaki et al. 2012a), and has additionally been found in two undescribed *henrichae* group species (Giblin-Davis et al. unpubl. obs.). The diagnostic utility of modified genital papillae has already been shown for another clade of Diplogastridae: in *Pristionchus* and the closely related genus *Parapristionchus* Kanzaki, Ragsdale, Herrmann, Mayer, Tanaka & Sommer, 2012, the v5 and v6 papillae are shrouded at the base by a socket-like structure, where the tip of v6 is split into two papilla-like projections (e.g., Kanzaki et al. 2012b, 2014a). Therefore, further examination of male tail morphology in other species of *Koerneria* sensu lato may confirm the importance of v5 and v6 as diagnostic of the *henrichae* group of *Allodiplogaster*. Yet another putative character for the *henrichae* group is the presence of a prerectum, as suggested to distinguish the “*Diplenteron* group” of *Koerneria* sensu lato (Fürst von Lieven 2008) and which was reported for *Allodiplogaster colobocerca* and *Allodiplogaster sudhausi*. The prerectum, which is a shallow but distinct constriction separating the posterior part of the intestine from the anterior part, can be found in three undescribed *Allodiplogaster* species (Kanzaki and Giblin-Davis unpubl. obs.), lending preliminary support to this idea. This character is not present in *Koerneria* nor in any species of *striata* group as interpreted from original descriptions, and therefore it may additionally support monophyly of the *henrichae* group.

## Footnotes

Dimorphism has been confirmed in at least two species in this group, including one undescribed species (Kanzaki and Ragsdale et al. unpubl. obs.)

## Supplementary materials

Supplementary material 1 Species status and several key characters of *Koerneria* sensu lato in the revised genera. Data type: Summary of typological characters as a spread sheet. Explanation note: Taxonomic history, morphological (typological) characters, and life-history traits of *Koerneria* sensu lato in the revised genera. This dataset is made available under the Open Database License (http://opendatacommons.org/licenses/odbl/1.0/). The Open Database License (ODbL) is a license agreement intended to allow users to freely share, modify, and use this Dataset while maintaining this same freedom for others, provided that the original source and author(s) are credited.Natsumi Kanzaki, Erik J. Ragsdale, Robin M. Giblin-Davis
